# Delir bei Demenz: Früherkennung und Behandlung – ein narrativer Review

**DOI:** 10.1007/s00391-022-02125-4

**Published:** 2022-10-27

**Authors:** Johanna De Biasi, Eva Reininghaus, Daniela Schoberer

**Affiliations:** 1 Medizinische Universität Graz, Graz, Österreich; 2 Universitätsklinik für Psychiatrie und Psychotherapeutische Medizin, Medizinische Universität Graz, Auenbruggerplatz 31, 8036 Graz, Österreich; 3 Institut für Pflegewissenschaft Graz, Graz, Österreich

**Keywords:** Überlagerndes Delir, Nichtpharmakologische Interventionen, Prävention, Diagnostik, Multiprofessionalität, Overlaying delirium, Non-pharmacological interventions, Prevention, Diagnosis, Multi-professionalism

## Abstract

**Hintergrund:**

Ein die Demenz überlagerndes Delir („delirium superimposed on dementia“, DSD) ist ein weit verbreitetes, aber häufig unerkanntes Problem. Ein solches Delir mindert die Lebensqualität von Patient/-innen mit einer Demenzerkrankung erheblich. Das Bewusstsein für dieses Zustandsbild muss bei Gesundheitsfachpersonen geschärft werden, um dessen Entwicklung frühzeitig erkennen und von Demenz ohne Delir unterscheiden zu können und dementsprechend zu behandeln.

**Ziel des Beitrages:**

Dieser Review beschreibt den bisherigen Stand der erforschten diagnostischen, präventiven und therapeutischen Methoden im Umgang mit DSD.

**Material und Methoden:**

Eine umfassende Literaturrecherche mit den Begriffen „Demenz“, „Delir“, „Delirium superimposed on dementia“ und „DSD“ wurde in der Datenbank PubMed durchgeführt, ebenso wie Literaturrecherchen über Google-Scholar.

**Ergebnisse:**

Die diagnostischen Möglichkeiten zur Detektion von DSD sind zurzeit auf Instrumente zur alleinigen Delirdiagnostik beschränkt, wie etwa die Confusion Assessment Method. Ein regelmäßiges multiprofessionelles Screening der Risikopatient/-innen ist zur frühen Detektion und Prävention dieser Erkrankung essenziell. Auch die Behandlung erfordert einen interdisziplinären Zugang. Eine pharmakologische Therapie ist indiziert, wenn nichtpharmakologische Maßnahmen nicht ausreichen oder zur Behandlung von delirauslösenden Ursachen. Im Vordergrund stehen die psychische und physische Betreuung der Patient/-innen und die Beseitigung von Risikofaktoren für die Entwicklung eines DSD.

**Diskussion:**

DSD ist ein ernst zu nehmendes Syndrom. Noch gibt es keinen Goldstandard in der Diagnostik und Therapie. Multikomponente nichtpharmakologische Interventionsprogramme reduzieren nachweislich die Inzidenz eines Delirs. Deren Nutzen bei DSD muss in großen multizentrischen Studien überprüft werden.

## Abstract (en)

**Background:**

Delirium superimposed on dementia (DSD) is a widespread but often undetected problem. Additional delirium significantly reduces the quality of life in patients with dementia. Awareness of this condition must be increased among professionals to recognize its development at an early stage, distinguish it from dementia and treat it accordingly.

**Objective:**

This review describes the current state of diagnostic, preventive, and therapeutic methods in dealing with DSD.

**Material and methods:**

A comprehensive literature search was conducted in the PubMed database using the terms “dementia”, “delirium”, “delirium superimposed on dementia” and “DSD”. An additional literature search was performed via Google Scholar.

**Results:**

The diagnostic techniques for detecting DSD are currently limited to delirium-only diagnostic tools, such as the confusion assessment method. A multiprofessional screening of high-risk patients on a regular basis is essential for the early detection and prevention of this disease. An interdisciplinary approach is also required for treatment. Pharmacological therapy is indicated when nonpharmacological measures are insufficient or to treat delirium-inducing causes. The focus lies on the mental and physical care of the patient and on the elimination of risk factors for developing DSD.

**Conclusion:**

DSD is a serious syndrome, however, there is still no gold standard for diagnosis and treatment. Multicomponent nonpharmacological intervention programs have been shown to reduce the incidence of delirium. Their benefit in DSD must be verified in large multicenter studies.

Das Delir ist eine akute fluktuierende neuropsychiatrische Erkrankung, die sich in einer Störung des Bewusstseins, Desorientierung, Aufmerksamkeits- und Wahrnehmungsstörungen äußert und bei den Betroffenen häufig zu erhöhter Morbidität und Mortalität führt. Tritt das Delir im Zusammenhang mit einer bereits bestehenden Demenz auf, wird es als „Delirium superimposed on dementia (DSD)“ bezeichnet. Die Diagnostik und Behandlung des DSD stellen im ambulanten und im klinischen Setting aus demografischen Gründen eine wachsende Herausforderung dar.

## Demenz, Delir und „Delirium superimposed on dementia“

Allein in Deutschland [1] und Österreich [2] leiden zurzeit (Stand 2021) etwa 1,7 Mio. Menschen an irgendeiner Form der Demenzerkrankung. Diese Zahl steigt jährlich um etwa 300.000 Neuerkrankte an [1]. Die Bezeichnung „Demenz“ wird laut Weltgesundheitsorganisation (WHO) und International Classification of Diseases (ICD-10) als Überbegriff verschiedener Subtypen derselben Krankheitsgruppe verwendet. Die Ursachen sind meist direkte Hirnschäden; häufig liegt ein neurodegenerativer Prozess zugrunde [3]. Das Erscheinungsbild der Demenz äußert sich in einem Rückgang höherer kortikaler Funktionen, begleitet von klinischen Verhaltensänderungen [4]. Die psychopathologische Symptomatik der Demenz kann bei betroffenen Patient/-innen ein auftretendes Delir maskieren [3]. Das Delir wird als multifaktoriell bedingtes, neuropsychiatrisches Syndrom mit akutem Ausbruch und fluktuierendem Verlauf definiert, man unterscheidet zwischen hyperaktivem, hypoaktivem und gemischtem Delir [5]. Die wichtigsten Risiko- bzw. auslösenden Faktoren sind hohes Alter, somatische Erkrankungen, Medikamente (v. a. Benzodiazepine), große Operationen, Entzug bei Substanzabusus (Alkoholentzugsdelir) und eine zugrunde liegende Demenzerkrankung [5, 6]. Neurodegenerative Prozesse im Alter, wie eine Demenzerkrankung, führen zu neuroinflammatorischen Zellveränderungen und Veränderungen der Konnektivität des Gehirns, wodurch das Gehirn die Fähigkeit verliert, adäquat auf einen akuten Stressor zu reagieren (=Delir) [7]. Umgekehrt begünstigt ein Delir auch die Entstehung und/oder Verschlechterung einer Demenzerkrankung. Eine stattgehabte Delirepisode erhöht bei älteren Patient/-innen das Risiko, eine Demenz zu entwickeln, um das 8Fache und führt nachweislich zu einem schlechteren kognitiven Outcome [8]. Es gibt eine direkte Assoziation zwischen einer Verschlechterung der Mini–Mental State Examination (MMSE) und der Länge und/oder Dauer eines Delirs. Neue Forschungen haben ergeben, dass die Ursache für eine neu auftretende Demenz nach einem Delir nicht Ablagerungen von β‑Amyloid und Tau-Proteinen sind, sondern neue Schäden am Gehirn sein können. Ob diese als Folge des Delirs auftreten oder parallel zum Delir durch delirauslösende Ereignisse entstehen, ist nicht geklärt [7].

### Prävalenz

Zahlen zur Prävalenz des DSD bei Patient/-innen über 65 Jahren mit einer Demenzerkrankung schwanken je nach Setting zwischen 22 und 89 % [9]. Die große Spanne in den Angaben zur Prävalenz ist dem geschuldet, dass ein Delir durch tageszeitliche Fluktuationen und den oft subsyndromalen Verlauf häufig nicht als solches erkannt wird. Laut klinischen Studien weisen beinahe bis zu zwei Drittel aller hospitalisierten über 65-Jährigen ein subsyndromales Delir auf [7]. Bei der Aufnahme in die Klinik haben bereits bis zu 25 % der Patient/-innen über 65 ein Delir; weitere 30 % entwickeln eines während des stationären Aufenthalts [10]. Demgegenüber wurde in mehreren Arbeiten erfasst, dass bei rund zwei Dritteln der hospitalisierten Delirpatient/-innen über 65-Jahren eine Demenzerkrankung zugrunde liegt, wobei das Risiko, ein Delir zu entwickeln, mit der Schwere der Demenzerkrankung steigt [3]. Mehrere Studien geben zudem an, dass die Delirprävalenz in Langzeitpflegeeinrichtungen (33,3–70,3 %) höher ausfällt als bei nichtinstitutionalisierten Patient/-innen [6]; etwas niedrigere Werte (9–57 %) wurden bei Patient/-innen in Kliniken festgestellt [7]. Die Werte schwanken je nach Ein- und Ausschlusskriterien (schwere Demenz, Einschränkungen in Sprache und Schrift) [6].

### Risikofaktoren und Folgen des DSD

Die Entwicklung eines Delirs bei vorbelasteten Patient/-innen ist abhängig von den komplexen Wechselbeziehungen zwischen prädisponierenden Faktoren (Vulnerabilität) und der Exposition gegenüber auslösenden Faktoren (Noxe). Bei Patient/-innen mit zugrunde liegenden Risikofaktoren (z. B. Demenz, Multimorbidität, Polypharmazie) können daher bereits relativ geringgradige Faktoren – z. B. ein leichter Harnwegsinfekt bzw. eine leicht bis mäßig ausgeprägte Elektrolytstörung – ausreichen, um ein Delir auszulösen [11]. Demenz mit überlagertem Delir wird mit höheren Kosten für das Gesundheitssystem, durchschnittlich längeren Krankenhausaufenthalten und schlechterem funktionellem Outcome als bei einer solitären Demenzerkrankung in Zusammenhang gebracht [9, 12]. Patient/-innen mit einer diagnostizierten Demenzerkrankung weisen während ihrer Behandlung in einer Einrichtung des Gesundheitswesens, ein etwa 3fach erhöhtes Risiko einer Delir-Entwicklung auf [13]. Dies erfordert eine genauere Beobachtung der betroffenen Risikogruppe, systematische Präventions- und Behandlungsmaßnahmen und v. a. ein geschultes Bewusstsein dieser Problematik von Gesundheitsfachpersonen. Ein Delir und insbesondere ein übersehenes und nichtbehandeltes Delir kann im weiteren Verlauf zu einem beschleunigten kognitiven Rückgang, zu verfrühter Einweisung in ein Pflegeheim und insgesamt zu höherer Morbidität und Mortalität führen [12]. Das Delir ist potenziell lebensgefährlich. Es kann jedoch bei frühzeitiger Diagnose behandelt werden, und mithilfe geeigneter präventiver Maßnahmen kann überhaupt das Risiko, ein solches zu entwickeln, reduziert werden. Nachfolgend werden Möglichkeiten der Diagnosestellung und Therapiekonzepte diskutiert.

### Methodik

Zur Identifizierung relevanter Literatur wurde eine Literaturrecherche in den Suchmaschinen PubMed und Google Scholar durchgeführt. Dabei wurden folgende Stichwörter mit Synonymen, jeweils in deutscher und englischer Sprache, angewandt: Delir, Demenz, DSD, Therapie, Prävention, Diagnostik, multikomponente Interventionsprogramme. Eingeschlossen wurden sowohl Studien, Berichte von Praxisprojekten sowie nationale und internationale Empfehlungen von Fachgesellschaften und Leitlinien, ohne zeitliche Beschränkung. Insgesamt wurden 231 Quellen gefunden, wovon, infolge des Titel‑, Abstract- und Volltextscreenings, 13 als relevant zur Diskussion der Identifizierung von Patient/-innen mit DSD und 7 als relevant zur Diskussion der therapeutischen Maßnahmen bei DSD befunden wurden.

## Wie wird ein DSD diagnostiziert?

**Figure 1:**
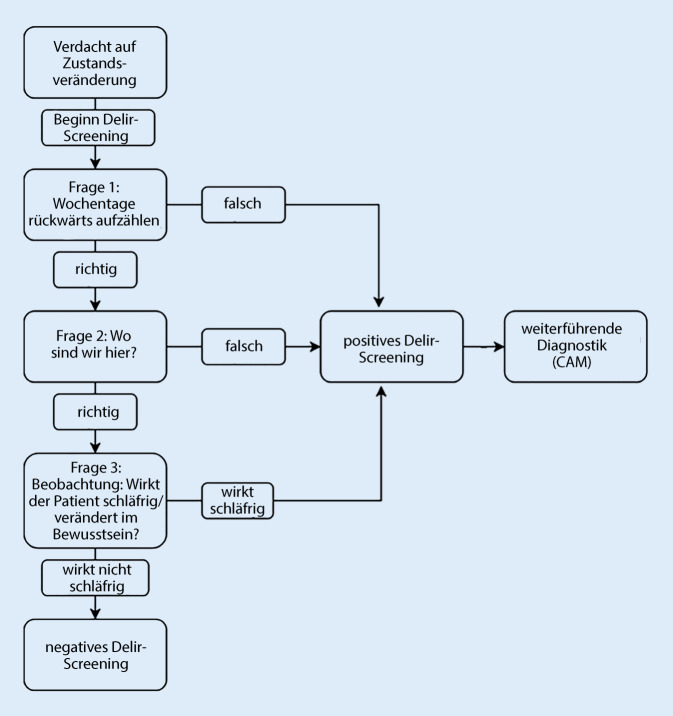

Die Österreichische Gesellschaft für Geriatrie und Gerontologie (ÖGGG) empfiehlt bei der Aufnahme in einer medizinischen Einrichtung ein standardisiertes Delirscreening bei allen Patient/-innen über 70 Jahren durchzuführen [5]. Die strukturierte Anwendung von Screeninginstrumenten soll eine frühzeitige Diagnostik unterstützen und gewährleisten, dass DSD bereits bei Patient/-innen mit zunächst asymptomatischem Erscheinungsbild entdeckt und rechtzeitig behandelt werden kann. In Ermangelung spezifisch entwickelter Tests zur Detektion des DSD werden in der Praxis zurzeit Instrumente verwendet, die zur Diagnostik des alleinigen Delirs entwickelt wurden [14]. Bereits im Jahre 1990 entwickelten Inouye et al. (1990) [15] ein Assessmentinstrument, das es Gesundheitsfachpersonen ermöglicht, ein Delir möglichst frühzeitig zu erkennen. Hierbei handelt es sich um die „Confusion Assessment Method“ (CAM). Noch heute ist dies eines der am meisten verwendeten Instrumente zur Diagnosesicherung eines Delirs [16]. In den Leitlinien der Universitätsklinik Jena wird die CAM als Goldstandard zur Delirdiagnostik genannt [17]. Ausgehend von der CAM wurde 2014 die 3D-CAM entwickelt; ein strukturierter Fragebogen, der eine einfache und schnelle Anwendung in der klinischen Praxis ermöglicht [18]. Die CAM und die 3D-CAM wurden jeweils in die deutsche Sprache übersetzt und validiert [19]. Steensma et al. (2019) [20] haben basierend auf den Fragen der 3D-CAM eine Kombination aus 3 Fragen mit hoher Sensitivität (94 %), aber begrenzter Spezifität (42 %) identifiziert, die dazu verwendet werden kann, bei bereits bekannter Demenz ein zusätzliches Delir schnell auszuschließen ([20]; Abb. 1).

Neben der CAM kommen im täglichen klinischen Setting die Kriterien der 5. Edition des „Diagnostic and Statistical Manual of Mental Disorders“ (DSM-5) und der ICD-10-Klassifikation zum Einsatz [21]. Anhand der DSM-5-Kriterien wurde die „Delirium Observation Scale (DOS)“ erstellt. Dieses Screening-Tool empfiehlt die Schweizer Leitlinie als geeignetes Instrument zur schnellen Erfassung eines Delirs [10]. Die schottische SIGN-Leitlinie (Scottish Intercollegiate Guidelines Network) nennt hingegen das 4AT-Tool (assessment test for delirium & cognitive impairment) zur Identifikation eines Delirs im akut-klinischen Rahmen [22]. Dabei werden Wachheit, Orientierung, Aufmerksamkeit und fluktuierende Symptomatik anhand eines Punktesystem bewertet und interpretiert [17]. Das 4AT-Tool liegt auch in validierter deutscher Form vor. Die erwähnten klinischen Instrumente wurden in erster Linie zur Erfassung des Delirs allein entwickelt und sind deshalb bei der Diagnostik eines DSD nur beschränkt aussagekräftig.

### Wie kann zwischen deliranter und demenzieller Symptomatik unterschieden werden?

2016 befragten Richardson et al. [21] 205 Delirspezialist/-innen zur Diagnostik des DSD. Die Befragten gaben an, dass Aufmerksamkeitsdefizit (71 %), Schwankungen des neurokognitiven Status (65 %) und Erregbarkeit (41 %) die wichtigsten Merkmale zur Diagnosesicherung eines Delirs im Unterschied zu „behavioural and psychological symptoms of dementia“ (BPSD) seien. Etwa drei Viertel der Befragten nannten „motorische Fluktuationen“ ebenfalls als maßgebendes Kriterium zur Bestimmung der DSD-Symptomatik [21]. Auch bei der Evaluierung des Screening-Tools „Recognizing Active Delirium as part of your Routine“ (RADAR), bestehend aus 3 Fragen, haben Voyer et al. (2016) [23] beobachtet, dass die Frage nach motorischen Verlangsamungen bei Personen mit DSD am häufigsten mit „Ja“ beantwortet wurde. In der Praxis werden jedoch kaum Tests zur Prüfung der motorischen Fluktuationen verwendet [21]. In mehreren Studien wurde die „Richmond Agitation Sedation Scale“ (RASS) als geeignetes Testverfahren zur Erkennung von motorischen Veränderungen beschrieben [21]. Die RASS ist eine Skala zur Beurteilung der Sedierung, des Bewusstseins und der Erregung, die ursprünglich auf der Intensivstation angewendet wurde. Als Basis-Tool zur schnellen Anwendung wurde eine modifizierte Version des RASS (mRASS) für das nichtintensivstationäre Setting entwickelt, die in < 30 s durchgeführt werden kann. Basierend auf diesen Erkenntnissen erarbeiteten Morandi et al. (2021) ein neues Tool zur spezifischen Diagnostik von DSD: das 4‑DSD [24]. Im 4‑DSD werden 4 Kriterien berücksichtigt: 1. Aktivitätsgrad (mRASS), 2. veränderte Hirnfunktion, 3. Aufmerksamkeit 4. akute Veränderung oder Schwankungen im mentalen Zustand. Die Kriterien werden anhand verschiedener Testverfahren evaluiert und mittels eines Punktesystems bewertet. Die Durchführung dauert im Durchschnitt 3 min. [24]. Die diagnostische Genauigkeit wurde in einer ersten Validitätstestung bei Patient/-innen mit einer moderat schweren Demenz als zufriedenstellend bewertet. Weitere psychometrische Testungen sind noch ausständig. Auch ist es aufgrund einer fehlenden validierten Übersetzung für die Anwendung im deutschen Sprachraum noch nicht geeignet.

Als Ergänzung zu spezifisch entwickelten Instrumenten zur Erfassung des DSD ist ein multifaktorieller und multiprofessioneller Zugang notwendig. Im Zusammenspiel soll ärztliches und nichtärztliches Personal bei Risikopatient/-innen ein regelmäßiges, im besten Fall tägliches Screening auf Delir durchführen. Dafür bietet sich ein validiertes Delirscreeninginstrument, wie die CAM, an.

## Welche therapeutischen Möglichkeiten gibt es?

Die wichtigsten Säulen im Management von DSD sind in erster Linie geeignete Präventionsmaßnahmen, frühzeitige Diagnose und Behandlung. Die verschiedenen Ausprägungen und Ursachen des DSD erschweren das Screening und machen es kaum möglich, ein allgemein gültiges Therapiekonzept zu entwickeln. In der Literatur werden verschiedene therapeutische Ansätze beschrieben und evaluiert. Angestrebte Therapieziele sind v. a. die Vermeidung von Komplikationen durch Hospitalisierung, die Behebung der zugrunde liegenden Erkrankung(en), die Verminderung kausaler Faktoren und die Unterstützung der Patient/-innen und deren Angehörigen [25]. Der Schwerpunkt liegt dabei auf nichtpharmakologischen supportiven Maßnahmen, u. a. auf der Überwachung des physiologischen Status und der psychosozialen Unterstützung der Patient/-innen im Rahmen von multikomponenten Interventionsprogrammen ([26]; Tab. 1). Pharmakologische Therapieansätze nehmen in der Behandlung eines DSD nur eine untergeordnete Rolle ein. Die beschriebenen therapeutischen Ansätze werden jeweils kurz angeschnitten.

**Table 1:** 

Therapie und Prävention	Beteiligte Berufsgruppen
Optimierung der klinischen Situation: d. h. Regulation von Flüssigkeits- und Elektrolythaushalt, Mangelernährung, Blutzucker, Blutdruck, Infektionen etc.	Arzt/Ärztinnen, Pflegefachpersonen
Absetzen delirogener Medikamente: besondere Überprüfung von vor dem Delir neu begonnenen Medikamenten	Arzt/Ärztinnen
Förderung der Orientierung, Anbieten von Seh- und Hörhilfe etc.	Pflegefachpersonen
Schlafhygiene (Licht, Lärm, Schlaf-Nacht-Rhythmus etc.)	Pflegefachpersonen
Vermeiden von Reizüberflutung/Reizdeprivation	Pflegefachpersonen
Frühmobilisation, unterstützende Ergo- und Physiotherapie	Ergo‑, Physiotherapeut/-innen
Engmaschige Betreuung und Ansprache, wenn möglich Bezugspflege	Pflegefachpersonen
Einbezug der Angehörigen (Rooming-in)	–
Symptomatische medikamentöse Therapie: Schmerzmanagement, Infektionen	Arzt/Ärztinnen

### Multikomponente, nichtpharmakologische Therapie: supportive Therapie zur Behandlung und Prävention von DSD

Im Vordergrund steht die Prävention eines Delirs. Um diese zu fördern, wurden strukturierte Delirpräventionsprogramme, wie das „Hospital Elder Life Program“ (HELP) entwickelt [27]. Dabei unterstützen sich Fachkräfte aus unterschiedlichen gesundheitlichen Sektoren in der Behandlung älterer Patient/-innen gegenseitig. Durch diese umfassende Betreuung kann die Delirrate nachweislich reduziert werden [27].

Besteht ein DSD, liegt der therapeutische Schwerpunkt auf der Behandlung der Grunderkrankung(en): Mangelernährung, Dehydratation, Schlafentzug sind häufige pathophysiologische Trigger für ein Delir [3]. Die therapeutischen Maßnahmen inkludieren die Regulation des Wasser- und Elektrolythaushalts, des Blutzuckers, des Blutdrucks und das Absetzen/Vermeiden von delirogenen Medikamenten [25]. Unterstützende Ergo- und Physiotherapie fördert die Autonomie der Patient/-innen und damit die Bewältigung grundlegender Aktivitäten im Alltag [3]. Wichtig sind ebenso eine beständige Betreuung, enger Kontakt zu den Angehörigen (ggf. Rooming-in) [28] und die Anpassung der Umgebung an die Bedürfnisse der Betroffenen. Ziel dabei ist es, Sicherheit im Alltag zu gewährleisten, Stürze zu verhindern, Schlafstörungen zu vermeiden, die Orientierung zu fördern, laute Geräusche, grelles Licht und exzessive Stimuli zu vermeiden, um die sensorische und wahrnehmungsbezogene Integrität zu erhalten [25]. Auch die Gewährleistung von Brillen und/oder Hörgeräten kann Verwirrtheitszuständen, die durch sensorische Wahrnehmungsstörungen hervorgerufenen sind, vorbeugen und somit das Risiko einer deliranten Symptomatik mildern. Basierend auf diesem Vorwissen wurden mehrere multikomponente nichtpharmakologische Interventionsprogramme, bestehend aus einer Kombination von Schmerzmanagement, Mobilisation, Schlafförderung, Ernährung, kognitiver und sensorischer Stimulation, entwickelt. Eckstein und Burkhardt veröffentlichten 2019 einen Scoping-Review zur Analyse von multikomponenten nichtpharmakologischen Interventionsprogrammen [26]. Von 25 eingeschlossenen Studien bezog sich nur eine ausschließlich auf Demenzpatient/-innen; zwei weitere Studien machten eine Subgruppenanalyse der Studienteilnehmer mit Demenz. Zwei dieser Studien verzeichneten einen signifikanten Rückgang der Delirinzidenz; die dritte registrierte eine signifikante Reduktion der Delirdauer. Aufgrund der geringen Anzahl an Subgruppenanalysen zu Kohorten mit zugrunde liegender Demenzerkrankung, lassen die Ergebnisse nur bedingt Schlussfolgerungen zu [26]. Große multizentrische Studien sind notwendig, um die Wirksamkeit multikomponenter nichtpharmakologischer Konzepte bei der Behandlung von DSD zu überprüfen.

### Pharmakologische Therapie

Ein evidenzbasiertes pharmakologisches Verfahren zur Behandlung des DSD ist noch nicht bekannt. Medikamentöse Maßnahmen müssen gegebenenfalls zur Behandlung eines delirauslösenden Prozesses eingesetzt werden, z. B. ein Antibiotikum zur Behandlung einer Infektion. 2019 veröffentlichten Burry et al. ein Cochrane-Review zur symptomatischen pharmakologischen Therapie eines Delirs [29]. Keine der 6 untersuchten Medikamentenklassen (Antipsychotika, α_2_-Agonisten, Statine, Opioide, Serotoninantagonisten, Cholinesterasehemmer) zeigte signifikante Vorteile in der Anzahl delirfreier Tage, in der Länge der Aufenthaltsdauer, im langfristigen kognitiven Outcome oder in der Mortalität [29]. Im Gegenteil, Polypharmakotherapie und viele Medikamentenklassen, die häufig bei älteren Personen zur Anwendung kommen (z. B. Anticholinergika), haben ein delirogenes Potenzial. Zusätzlich sind Personen mit Demenz noch vulnerabler für somatische Nebenwirkungen (Obstipation, Harnverhalt etc.), welche wiederum ein Delir begünstigen [5].

## Fazit für die Praxis

- DSD verläuft häufig lebensbedrohlich und kann bei den Betroffenen zu hohem Leidensdruck und Einschränkungen im Alltag führen. Wegen seiner unspezifischen Symptomatik bleibt das Syndrom häufig lange unentdeckt.
- Delir und Demenz beeinflussen sich gegenseitig. Das eine begünstigt jeweils das Auftreten und/oder die Verschlechterung des anderen.
- Mit geeigneten Instrumenten sollte ein regelmäßiges Screening auf Delir bei Risikopatient/-innen durchgeführt werden.
- Ein multiprofessioneller und interdisziplinärer Zugang ist sowohl in der Diagnostik als auch in der Behandlung eines DSD wichtig.
- Multikomponente nichtpharmakologische Interventionsprogramme scheinen ein vielversprechender Therapieansatz zu sein. Um deren Wirksamkeit bei DSD zu überprüfen, sind große multizentrische Studien notwendig.
